# Functional Exploration Of T-Type Calcium Channels (Cav3.2 And Cav3.3) And Their Sensitivity To Zinc

**DOI:** 10.2174/1874285801812010280

**Published:** 2018-07-31

**Authors:** Tahar Hazzaz Abouamal, Zineb Choukairi, Fechtali Taoufiq

**Affiliations:** Department of Biology, Laboratory of Biosciences, Faculty of Sciences and Technology of Mohammedia, Casablanca, Morocco

**Keywords:** Voltage gated channel, t-type calcium channel, Electrophysiology, Patch Clamp, Perferential Blocker, Electrophysiological

## Abstract

**Introduction:**

T-type Ca^2+^ channels (TTCC) are low Voltage-gated calcium channels, expressed in various tissues such as the brain and heart, and contribute to a variety of physiological functions including neuronal excitability, hormone secretion, muscle contraction, and pacemaker activity. At high concentrations, Zinc (Zn^2+^) is naturally attached to cell membranes and is therefore considered a reversible inhibitor of calcium. Zinc is also involved in the kinetics of sodium and potassium currents. Zinc is essential for many functions. A low zinc tenor is associated with emotional instability, digestive disorders, slow-growing and alteration of protein synthesis.

**Material and Methods:**

For the Cell Culture we used HEK-293/tsA-201, and for transfection, the pCDNA3 plasmid constructs encoding human CaV3.2, and CaV3.3 subunits. Electrophysiological experiments were performed using the whole cell configuration of the patch-clamp technique. T-type currents were recorded using a test pulse from a holding potential at (-100mV) to (-30 mV), data Acquisition and Analysis for Current-voltage relationships (I-V curves) were recorded for the two cloned T-type Ca^2+^ channels (Cav3.2, Cav3.3).

**Results:**

Our studies describe the behavior of these channels Cav3.2 and Cav3.3 and also their current sensitivity to Zinc (Zn^2+^) in transfected HEK-293/tsA-201cells. Our results show that Zn^2+^ applies a modulatory effect on T-type calcium channels. We observe that Zn^2+^ differentially modulates the CaV3.2 and CaV3.3 channels. Zn^2+^ preferably inhibits Cav3.2.

**Conclusion:**

We have demonstrated that Zn^2+^ differentially modulates two CaV3 channels (Cav3.2 and Cav3.3): It is a preferential blocker of CaV3.2 channels and it alters the gating behaviour of CaV3.3 channels.

## 1 INTRODUCTION

Gated calcium channels are present in various cells of the human body and their pharmacological properties are independent of cell type where they reside [1].

T-type channels have distinct functional properties compared to L-type channels: Faster kinetics of inactivation and activation, and slower deactivation kinetics. Since T channels are able to activate by low depolarization near resting potential of the membrane, their functions are also related to the control of neuronal excitability [2-6]. T-type Ca^2+^ channels (TTCC) are low Voltage-gated calcium channels, expressed in various tissues such as the brain and heart, and contribute to a variety of physiological functions including neuronal excitability, hormone secretion, muscle contraction, and pacemaker activity. In addition, an increasing body of evidence shows that TTCC can trigger fast and low-threshold exocytosis [7-14].

On the other hand, Zinc is essential for all mammals because it has various actions on nerve cells. However, it is widely reported that zinc can be very toxic to neurons in small amounts. At high concentrations, zinc (Zn^2+^) is naturally attached to cell membranes and is therefore considered a reversible inhibitor of calcium.

Zinc is also involved in the kinetics of sodium and potassium currents. Zinc is essential for many functions. A low zinc tenor is associated with emotional instability, digestive disorders, slow-growing and alteration of protein synthesis.

Despite the fact that Zn^2+^ is related to proteins in biological systems, it is also involved in some neurodegenerative diseases such as Alzheimer's disease [15], and synaptic plasticity [16], amyotrophic lateral sclerosis [17] and Parkinson's disease [18]. Zinc is also involved in some neuropathology [19-23].

## 2 METHODS

Cell Culture and Transfection - HEK-293/tsA-201 cells were cultivated in Dulbecco’s modified Eagle’s medium supplemented with glutamax and 10% fetal bovine serum (Life technologies) using standard techniques.

For transfection, we use pCDNA3 plasmid constructs encoding human CaV3.2 [24], and CaV3.3 subunits [25]. We also use NaCl (150 mM) and the transfection agent Jet-PEI (QBiogen, Illkirch, France). The green fluorescent protein (GFP) was co-transfected to monitor cells.Cells were grown to 90% confluence in 35-mm Petri dishes. 36-48h after transfection, GFP - positive cells expressing sufficient levels of the Ca^2+^ channels were examined for electrophysiological recordings. To this end, cells were split by trypsin, plated on petri dishes at 510% confluence with 2 ml of mix (SVF + DMEM), and given 2 - 3 h to settle before starting the patch clamp.

## 2.1 Preparation of Intracellular and Extracellular Media

 The preparation of the intracellular and extracellular media are necessary to start the patch clamp experiments. The extracellular solution contained (in mM): 135 NaCl, 20 TEA-Cl and 2 CaCh, 1 MgCl2, 10 HEPES pH to 7, 4 The internal pipette solution contained (in mM): 140 CsCl, 10 EGTA, 3 CaCh, and 10 HEPES ph to 7,2.

## 2.2 Electrophysiology

Electrophysiological experiments were performed using the wholecell configuration of the patch-clamp technique. Recordings were obtained using an Axopatch 200 and pCLAMP data acquisition software (Axon Instruments, Inc., Union City, CA, USA). The sampling frequency for acquisition was 10 kHz and data were filtered at 2 kHz. Zinc chloride (ZnCl2) was purchased from Sigma-Aldrich (France), and was dissolved in extracellular medium at 1 M as a stock solution, kept at 4°C and applied at final concentrations to recorded cells by a gravity-driven perfusion device controlled by solenoid valves.

## 2.3 Patch Clamp Recordings on Cultured Cells

In HEK-293 cells, T-type whole-cell patch clamp recordings were performed 2-3 days after transfection. T-type currents were recorded using a test pulse from a holding potential at (-100mV) to (-30 mV) and using 2 mM Ca^2+^ at room temperature. The current density was calculated according to the capacitance of the cell and expressed in pA/picofarad.

## 2.4 Data Acquisition and Analysis 

 Current-voltage relationships (I-V curves) were recorded for the two cloned T-type Ca^2+^ channels (Cav3.2, Cav3.3), The protocol stepped the cell membrane potential from -100 mV to test potentials starting at -100 and increasing to +70 mV in 10 mV or 5 mV increments. For Zn^2+^, we use the protocol Test potential from -100 mV to -30 mV and 10 step for each concentration between (0pM and 100pM).

## 3 RESULTS

### 3.1 Records of Calcium T-type Ca^2+^ Channels

#### 3.1.1 For Cav 3.2

The results obtained in this studyfor CaV3.2 showed that the biophysical behavior T-type calcium channel varies with the applied voltage. We obtained values from the software pCLAMP™ and Clampfit 10.7.0 for data analysis.

The potential values are going from -80mV to +70 mV and a current: From -30.639 pA at (-80mV) to - 786.132pA at (-35 mV) and then to 26.489pA at (+70mV), the Fig. (**1A**) shows the currents of calcium channels CaV3.2, The red line show the first Sweep (recording 1), All data from recording are summarized in (Table **1**). below:

#### 3.1.2 For Cav 3.3

The results obtained in this study, for CaV3.3 showed that the biophysical behavior T-type calcium channel varies with the applied voltage. We obtained values from the software pCLAMP™ and Clampfit 10.7.0 for data analysis.

The potential values are going from -100mV to +70 mV and a current: From -15.136718pA at(-100mV) to -515.86pA at (-30 mV) and then to 44.67pA at (+70mV), the Fig. (**1B**) shows the currents of calcium channels CaV3.3, The red line show the first Sweep (recording 1), all data from recording are summarized in (Table **2**). below:

### 3.2 Zn^2+^ Inhibition of T-Type Calcium Channels

To determine the molecular consequences of the Zinc (Zn^2+^) on channel function. The biophysical properties of the channels were characterized by standard whole-cell patch clamp techniques.We recorded macroscopic Ca^2+^ current in the whole-cell configuration to define the effect of Zinc (Zn^2+^) on cloned channels (Cav3.2 and Cav3.3).

Fig. (**2A**-**2B**) shows the recording of zinc on calcium channels 3.2 and 3.3, The space between the bold curves represents the change in concentration between each 10 sweeps, The level of inhibition is proportional to the concentration of Zn2+. When zinc concentration is increased, the level of inhibition also increases.

The red line in Fig. (**2A** show the Sweep 26 with voltage of: -35.2783 pA and 100 uM of Zn2+ Concentration. 

The red line in Fig. (**2B**) show the Sweep 26 with voltage of: -542.236 pA that represent the first sweep after washout.

The error bars in Fig. (**2C** and **2D**) represent the SE (standard error); For exemple for CAV3.2 the I/Ictrl is between 0,724971 and 0,9652457 for Log [Zn^2+^] = -7 (0,1 µM) with n = 10 and between 0,504065 and 0,7732869 for Log[Zn2^+^] = - 6 (1µM) with n = 8.For CAV3.3 the I/Ictrl is between 0,876818 and 0,9874821 for Log [Zn^2+^] = -6 (1 µM) with n = 10 and between 0,8043191 and 0,9179184 for Log [Zn^2+^] = - 5 (10 µM) with n = 10.

For CAV3.3 the I/Ictrl is between 0,876818 and 0,9874821 for Log [Zn^2+^] = -6 (1 µM) with n = 10 and between 0,8043191 and 0,9179184 for Log [Zn2^+^] = - 5 (10 µM) with n = 10.

The Fig. (**2E**) shows a bar graph of the average inhibition of CaV3.2 and CaV3.3 currents with 1 µM Zn^+^.

Fig. (**2F**) shows the dose response relationships for Zn^2+^ inhibition of CaV3.2 and CaV3.3 currents. The fraction of unblocked peak current (I/ICtrl) is plotted against Zn2^+^ concentration. The IC50 values were obtained from fitted data using a sigmoidal dose response with variable Hill slope equation with results for CAV3.2 (IC_50_ = 0.7648) and for CAV3.3 (IC_50_ = 0.5097)

The detailed data for the sigmoidal dose-response are present in (Table **3**) below:

## 4 DISCUSSION

In the present study, we observe that Zn^2+^ differentially modulates the CaV3.2 and CaV3.3 channels. Zn^2+^ preferably inhibits Cav3.2 channels with an IC50 in the submicromolar range (~ 0.8 pm), which is 100 to 200 times lower than that of CaV3.3 channels. Zn^2+^ inhibition of CaV3 channels is related to a negative change in steady state activation and inactivation properties, except CaV3.2 steady state activation properties which remain the same.

What we can conclude from our study is that Zn^2+^ involves a significant slowdown in the inactivation kinetics of the CaV3.3 currents, as well as a very apparent slowdown in CaV3.3 current deactivation kinetics. As a result, it has been found through action potential clamping experiments in cells that express CaV3.3, that Zn^2+^ can cause a significant increase in Ca2 + input, especially during the potential range of post depolarizing.

## CONCLUSION

In conclusion, we have demonstrated that Zn^2+^ differentially modulates two CaV3 channels (Cav3.2 and Cav3.3): it is a preferential blocker of CaV3.2 channels and it alters the gating behaviour of CaV3.3 channels.

## ETHICS APPROVAL AND CONSENT TO PARTICIPATE

Not applicable.

## HUMAN AND ANIMAL RIGHTS

 No animals/humans were used for studies that are the basis of this review.

## CONSENT FOR PUBLICATION

Not applicable.

## CONFLICT OF INTEREST

 The authors declare no conflict of interest, financial or otherwise.

## ACKNOWLEDGEMENTS

Declared none.

## Figures and Tables

**Fig. (1):**
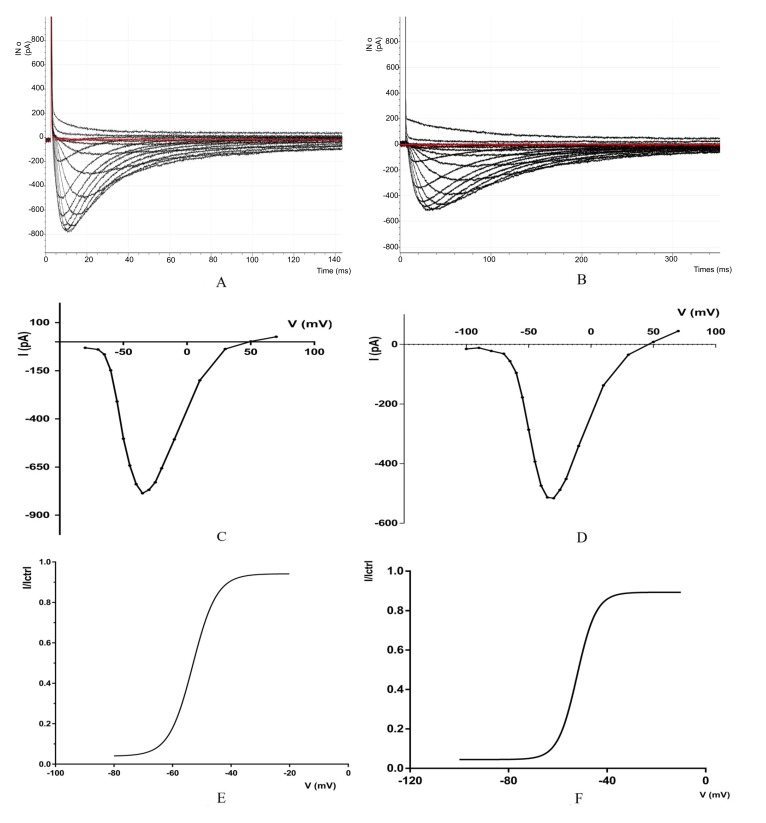
**Family of control CaV3.2 and CaV3.3 currents** **(A)** Recoding of calcium channels CAv3.2 with IV protocol the selected current trace correspond to the following depolarizing voltage pulses (mV): - 80, -70, -65, -50, -45, -40, -35, (peak current), -30, -25, -20, -10, -10, -30, -50, -70 from a HP of -100 m V. **(B)** Recording of calcium channels CAv 3.2 with IV protocol, the selected current traces correspond to the following depolarizing voltage pulses (mV: - 100, -90, -80, -70, -65, -60, -55, -50, -45, -35, -30 (peak current), -25, -20, -10, 10, -30, -50, 70, from a HP of -100, -90, -80, -70, 65, -60, -55, -80, -45, -40, -35, -30 (peak current), -25, -20, -10, 10, 30 50, 70, from a IP of -100mV. (CD Mean current -voltage (1-V)) relationships of CaV3.2 **(C)** and CaV3.3 **(D)** currents. The HP of - 100 mV. (E.F) steady-state activation curves were obtained from the fitting of the 1-V curves. Steady -state inactivation for CaV3.2 **(E)** and CaV3.3 **(F)** currents.

**Fig. (2):**
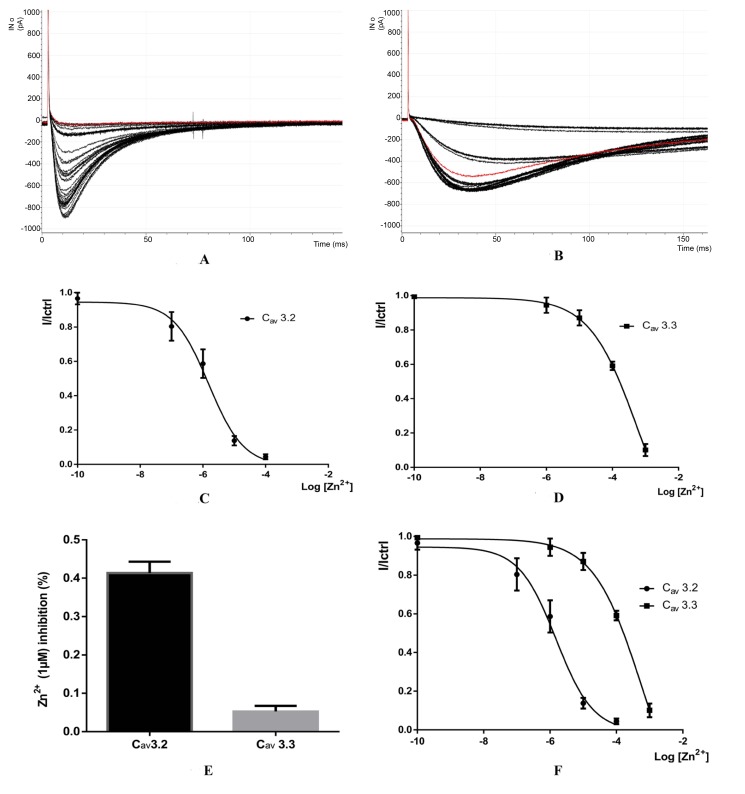
**Zinc inhibits human CaV3.2 and CaV3.3 T-Type Ca2^+^ channels** **(A-B)** Recording calcium channels CaV3.2 and Cav3.3 after application of 0.1 µM, 1 µM, 10µM and 100µm of Zinc (Zn2^+^), **(C-D)** Dose - response for Zn2^+^) injibition of CaV3.2 and CaV3.3, Fraction of unblocked peak current (1/1Ctrl) is plotted against Zn2^+^ concentration. The IC_50_ values were obtained from fitted data using a sigmoidal does-response with variable Hill slope equation **(e)** Bar graph of the average inhibition of Cav3.3 currents by 1 µM Zn2^+^ **(F)**, dose-response relationships for Zn^2+^ inhibitaion of caV3.2 and CaV3.3 currents.

**Table 1:** Records of calcium T-type Ca^2+^ channels for Cav3.2.

**Voltage (mV)**	-80	-70	-65	-55	-50	-45	-40	-35
**Current (pA)**	-30.639	-39.550	-64.941	-309.570	-502.807	-641.967	-738.769	-786.132
**Voltage (mV)**	-30	-25	-20	-10	10	30	50	70
**Current (pA)**	-768.188	-729.003	-656.738	-506.347	-199.829	-36.865	2.197	26.489

**Table 2:** Records of calcium T-type Ca2^+^ channels for Cav 3.3.

**Voltage (mV)**	-100	-90	-80	-70	-65	-55	-50	-45
**Current (pA)**	-15,136	-11,596	-21,972	-31,127	-56,030	-177,734	-286,132	-393,188
**Voltage (mV)**	-40	-35	-30	-20	-10	10	30	50
**Current (pA)**	-473,998	-513,183	-515,86	-451,538	-340,820	-137,573	-34,423	8,300

**Table 3:** Sigmoidal dose-response for Cav 3.2 and Cav 3.3.

Sigmoidal Dose-Response	Cav 3.2 av	Cav 3.3 av
**Sigmoidal, 4PL, X is log(concentration)**	–	–
**Best-fit values**	–	–
**Top**	0,9451	0,9874
**Bottom**	-0,00867	-0,5418
**LogIC50**	-5,819	-3,234
**HillSIope**	-0,7779	-0,5984
**IC50**	0,000001519	0,0005834
**Span**	0,9537	1,529
**Std. Error**		
**Top**	0,01934	0,01131
**Bottom**	0,03031	0,2655
**LogIC50**	0,06923	0,2627
**HillSlope**	0,08143	0,07248
**Span**	0,03957	0,272
**95% Confidence Intervals**		
**Top**	0,9061 to 0,9841	0,9645 to 1,010
**Bottom**	-0,06980 to 0,05246	-1,078 to -0,005667
**LogIC50**	-5,958 to -5,679	-3,764 to -2,704
**HillSIope**	-0,9421 to -0,6137	-0,7447 to -0,4520
**IC50**	1,101e-006 to 2,094e-006	0,0001720 to 0,001979
**Span**	0,8739 to 1,034	0,9799 to 2,078
